# Current status of smallpox vaccine.

**DOI:** 10.3201/eid0504.990429

**Published:** 1999

**Authors:** J W LeDuc, J Becher


### Current Status of Smallpox Vaccine

**To the Editor:** The possible use of smallpox virus as a weapon by terrorists has stimulated growing international concern and led to a recent review by the World Health Organization of the global availability of smallpox vaccine. This review found approximately 60 million doses worldwide, with little current vaccine manufacture, although limited vaccine seed remains available (1). Ongoing discussions in the United States suggest that the national stockpile should contain at least 40 million doses to be held in reserve for emergency use, including in case of a terrorist release of smallpox virus (O'Toole, this issue, pp. 540-6).

The current U.S. stockpile contains approximately 15.4 million doses of vaccinia vaccine (Dryvax) made from the New York City Board of Health strain of vaccinia and was produced by Wyeth Laboratories in 13 separate lots. The vaccine is lyophylized in glass vials with rubber stoppers and sealed with a metal band. When rehydrated, each vial contains 100 doses and has a potency of at least $10^8$ plaque-forming units (pfu)/ml. Some vials of the vaccine stockpile have shown elevated moisture levels and thus failed routine quality control testing; however, the vaccine in these vials remains potent, and the failed lots have not been discarded.

The diluent used to rehydrate the vaccine contains brilliant green, which makes the vaccine easier to visualize when administered with bifurcated needles. Over time, the brilliant green has deteriorated, and most of the available diluent does not pass quality control. Discussions are under way with Wyeth to begin production of sufficient new diluent for the entire stockpile.

The vaccine is administered by superficial inoculation (scarification) with a bifurcated needle. Fewer than 1 million bifurcated needles are held as part of the stockpile. As with the diluent, Wyeth has been requested to produce additional bifurcated needles.

Vaccinia virus produces adverse reactions in a small percentage of vaccinated persons. Adverse reactions are treated with vaccinia immune globulin (VIG), currently only available from Baxter Healthcare Corporation (5,400 vials of VIG in stock). Each vial contains 5 ml of VIG; the recommended dose for postvaccine complications is 0.6 ml per kg of body weight. This volume is sufficient to treat adverse reactions in approximately 675 adults. Further, the entire stockpile of VIG has been placed on hold while the cause of a slight pink discoloration is investigated. Until the cause of the discoloration is determined or another approved supply of VIG is obtained, no vaccinia vaccine is being released. While unknown, the rate of adverse reactions in today's population is likely to be greater than seen during the global eradication campaign because of recent increases in the number of immunocompromised persons. The Department of Defense has recently contracted the processing of new lots of VIG (to be administered intravenously rather than by the intramuscular route like existing VIG stocks); however, maintaining adequate stocks of VIG will remain a challenge.

In the event of release of smallpox virus, persons at high risk and persons exposed but not yet showing clinical illness would be vaccinated immediately. Intensive case detection and vaccination of contacts and other persons at risk would follow. All vaccine, including lots retained after failed quality control tests, would be made available for emergency use. Previous studies have found that more than 90% of susceptible persons respond to vaccinia virus with a titer of $10^7$ pocks/ml (2). In an emergency, consideration would be given to diluting the existing vaccine as much as 10-fold, so that each vial could conceivably contain 1,000 doses of vaccine, rather than the current 100 doses. The present vaccine container is sufficiently large to accommodate the added diluent. The absence of sufficient quantities of VIG to protect against adverse reactions during a mass immunization campaign would necessitate careful screening of those receiving the vaccine; some persons with adverse reactions would likely go untreated.

While the intentional release of smallpox virus would represent a global emergency, the existing national stockpile could be effectively used to limit the spread of disease and buy time while the pharmaceutical industry begins emergency vaccine production.

**James W. LeDuc and John Becher**
Centers for Disease Control and Prevention, Atlanta, Georgia, USA

### References

1. World Health Organization. Report of the meeting of the ad hoc committee on Orthopox virus infections. Department of Communicable Disease Surveillance and Response. WHO, 14-15 January 1999.
2. Cockburn WC, Cross RM, Downie AW, Dumbell KR, Kaplan C, Mclean D, et al. Laboratory and vaccination studies with dried smallpox vaccines. Bull World Health Organ 1957;16:63-77.

### West Nile Fever in Czechland

**To the Editor:** After heavy rains in July 1997, extensive floods occurred along the Morava River, Czech Republic. Populations of *Aedes* mosquitoes increased rapidly in the flooded areas, prompting surveillance for mosquito-borne virus infections in the Breclav area, South Moravia. We collected 11,334 female mosquitoes (9,100 *Aedes vexans,* 917 *Ae. cinereus,* 11 *Ae. cantans,* 1,074 *Ae. sticticus*, and 232 *Culex p. pipiens*) from July through September 1997 and tested them for virus in 117 monospecific pools by intracranial inoculation of suckling mice. Seven virus isolates were obtained and identified by complement-fixation and neutralization tests. Six isolates (five from *Ae. vexans*, one from *Ae. cinereus*) were identified as the bunyavirus Tahyna, California serogroup, and one (strain 97-103 from 57 *C. p. pipiens* collected at Lanzhot, 48°40'N, 16°56'E, on September 17) was identified as the flavivirus West Nile (1). A crossed comparison of 97-103 and topotype Eg-101 (2) West Nile virus strains and their antisera (prepared in mice by three intraperitoneal doses at weekly intervals) by plaque reduction neutralization (PRN) on XTC-2 cells (3,4) showed their antigenic relationships: reciprocal titers of homologous/heterologous sera were 512/512 in Eg-101 and 512/64 in 97-103. Strain 97-103 has lower virulence than Eg-101 in that it does not kill adult ICR mice and may represent a subtype of West Nile virus.

Blood samples were obtained from 619 persons seeking treatment at hospital and outpatient clinics in the Breclav area from June 23 through September 29, 1997. Sera were inactivated at 56°C for 30 minutes, diluted 1:8, and assayed by PRN for antibodies against *c.* 30 plaque-forming units (PFU) per well of West Nile virus strains Eg-101 and 97-103. All sera causing 90% reduction of PFU at 1:8 dilution were titrated, and the highest serum dilution showing 50% PFU reduction was regarded as the titer. Antibodies neutralizing West Nile virus were detected in 13 (2.1%) persons: 2.8% of 179 male and 1.8% of 440 female. Persons with detectable West Nile virus antibody were questioned about their health history during the previous 5 years, and their medical records were reviewed; none recalled having had tickborne encephalitis (Central-European encephalitis [CEE] virus is the only other flavivirus present in Czechland) or having been vaccinated against CEE or yellow fever virus. Titers of PRN antibodies to CEEV were all below 16. Two of the seropositive persons had traveled abroad during the last 5 years: one to Croatia in 1996, and one to South Australia during 1951 to 1994.

Paired serum samples were obtained from 72 of the 619 persons examined. A significant increase (≥4 times) in antibody titer against West Nile virus between the first (acute-phase) and second (convalescent-phase) samples was detected four times: in 2 of 41 young persons (≤16 years of age) and in 2 of 31 adults (>16 years of age). Among the four seroconverting persons, only the two children had clinical symptoms compatible with West Nile fever. A 9-year-old boy had fever (39°C) for 4 days, sore throat, headache, muscle ache, pronounced fatigue, and nausea lasting approximately 6 days, with recovery after 13 days. Neutralizing antibodies to West Nile virus, Eg-101 and 97-103, were 64 and 32 on July 22 and 512 and 256 on August 4, respectively. A 9-year-old girl had fever (38°C-39°C) for 3 days, sore throat, headache, muscle ache, pronounced fatigue, nausea, vomiting, maculopapular rash (including flushed face), and slightly enlarged inguinal lymph nodes. The illness lasted approximately 7 days, with complete recovery after 17 days. Neutralizing antibodies to West Nile virus, Eg-101 and 97-103, were 64 and 32 on August 6 and 256 and 128 on August 20, respectively. Of the remaining nine seropositive persons lacking paired serum samples, one had severe headache, muscle ache, prolonged fatigue, nausea, pain on eye movement, maculopapular rash, and insomnia in summer of 1997. Two other persons had had "summer fever" (sore throat and lymphadenitis; headache with pain on eye movement) in 1997. The other persons who seroconverted did not report any substantial illness. In total, clinical symptoms in five persons are compatible with West Nile fever.

